# Knowledge of Acquired Immune Deficiency Syndrome (AIDS) and Syphilis Among Education Faculty Students: A Text Mining Analysis

**DOI:** 10.7759/cureus.103460

**Published:** 2026-02-12

**Authors:** Chisako Okai, Akira Yoshioka, Nobuhiro Nasu, Akihiro Yokoyama, Hiromi Suzuki, Rumi Nohara, Yasuko Maekawa, Hiroe Kitamura, Natsuki Shimizu, Nobuyuki Miyatake

**Affiliations:** 1 Department of Hygiene, Faculty of Medicine, Kagawa University, Miki, JPN; 2 Faculty of Education, Kansai University of Social Welfare, Hyogo, JPN; 3 Department of Physical Therapy, Okayama Healthcare Profassional University, Okayama, JPN; 4 Department of Hygiene, Faculty of Medicine, Kagawa University, Kagawa, JPN; 5 Department of Nursing, Faculty of Medicine, Kagawa University, Miki, JPN; 6 Faculty of Nursing, Kansai University of Social Welfare, Ako, JPN

**Keywords:** aids, faculty of education, sexually transmitted infections (stis), syphilis, text mining analysis

## Abstract

Objective: The proper management of sexually transmitted infections (STIs), such as acquired immune deficiency syndrome (AIDS) and syphilis, has become a public health challenge in Japan and worldwide. We herein examined knowledge of AIDS and syphilis among educational faculty students closely involved in future sex education using a text mining analysis.

Methods: A total of 126 (74 males and 52 females, 19.1 ± 0.1 years) of 176 eligible first- and second-year educational faculty students at a university in Hyogo Prefecture, Japan, were enrolled. We conducted open-ended surveys asking participants to describe what they knew about AIDS and syphilis in written responses. We also investigated participants’ knowledge of the number of AIDS and syphilis patients in Japan, their sexual intercourse experience, use of contraceptives for infection prevention, and fears toward AIDS and syphilis.

Results: Fifty males (67.6%) and 28 females (53.9%) had sexual intercourse experience. Among these, 76% of males and 69% of females consistently used contraceptives. Sixty-four males (86.5%) and 48 females (92.3%) were afraid of AIDS and syphilis. The text mining analysis showed that the total number of words was 466 for AIDS and 325 for syphilis, indicating fewer words for the latter. An analysis using co-occurrence networks also showed fewer word types and clusters for syphilis than for AIDS.

Conclusion: Knowledge of AIDS and syphilis, particularly syphilis, appears to be limited among first- and second-year educational faculty students. Due to their future involvement in sex education, the implementation of appropriate and fundamental education regarding STIs is necessary.

## Introduction

Sexually transmitted infections (STIs), including acquired immune deficiency syndrome (AIDS) and syphilis, have become a public health challenge in Japan and worldwide. The number of AIDS patients has slightly decreased in the past decade [1], whereas the number of syphilis patients has markedly increased [2]. For example, 960 AIDS patients and 15,055 syphilis patients have reported by the Ministry of Health, Labour and Welfare, Japan in 2023 [1,2]. Therefore, the proper prevention and management of AIDS and syphilis, particularly syphilis, are urgently required in Japan.

Sex education in Japan is covered under the prevention of infectious diseases within the health section of the junior high school curriculum guidelines [3]. Educational faculty students will be involved in children’s sex education in the future and will play an important role in sex education. Research on knowledge and motivation regarding STIs among students aspiring to become teachers has been conducted, demonstrating the importance of not only early education but also ongoing education [4].

Studies on STIs, such as AIDS and syphilis, in young adults, including university students [5-7], mainly conducted a quantitative analysis using multiple-choice questionnaires, while only a few performed a qualitative analysis using text mining, particularly in Japan. These studies generally reported insufficient knowledge about STIs, low risk perception, and inconsistent preventive behaviors among university students. We previously reported perceptions of coronavirus disease 2019 (COVID-19) vaccination [8-11] and human papillomavirus vaccination [12] in Japan. We also investigated perceptions of AIDS and syphilis among healthcare professional university students with a physical therapy course, and found that the term “be cured” was a characteristic word for AIDS, while “frightening” was common for syphilis in females and for AIDS in males [13].

Therefore, we herein examined knowledge of AIDS and syphilis in educational faculty students using qualitative analyses, such as text mining. To investigate the content of sex education for educational faculty students at universities, a survey primarily targeting first- and second-year students was conducted.

## Materials and methods

Subjects

**Figure 1:**
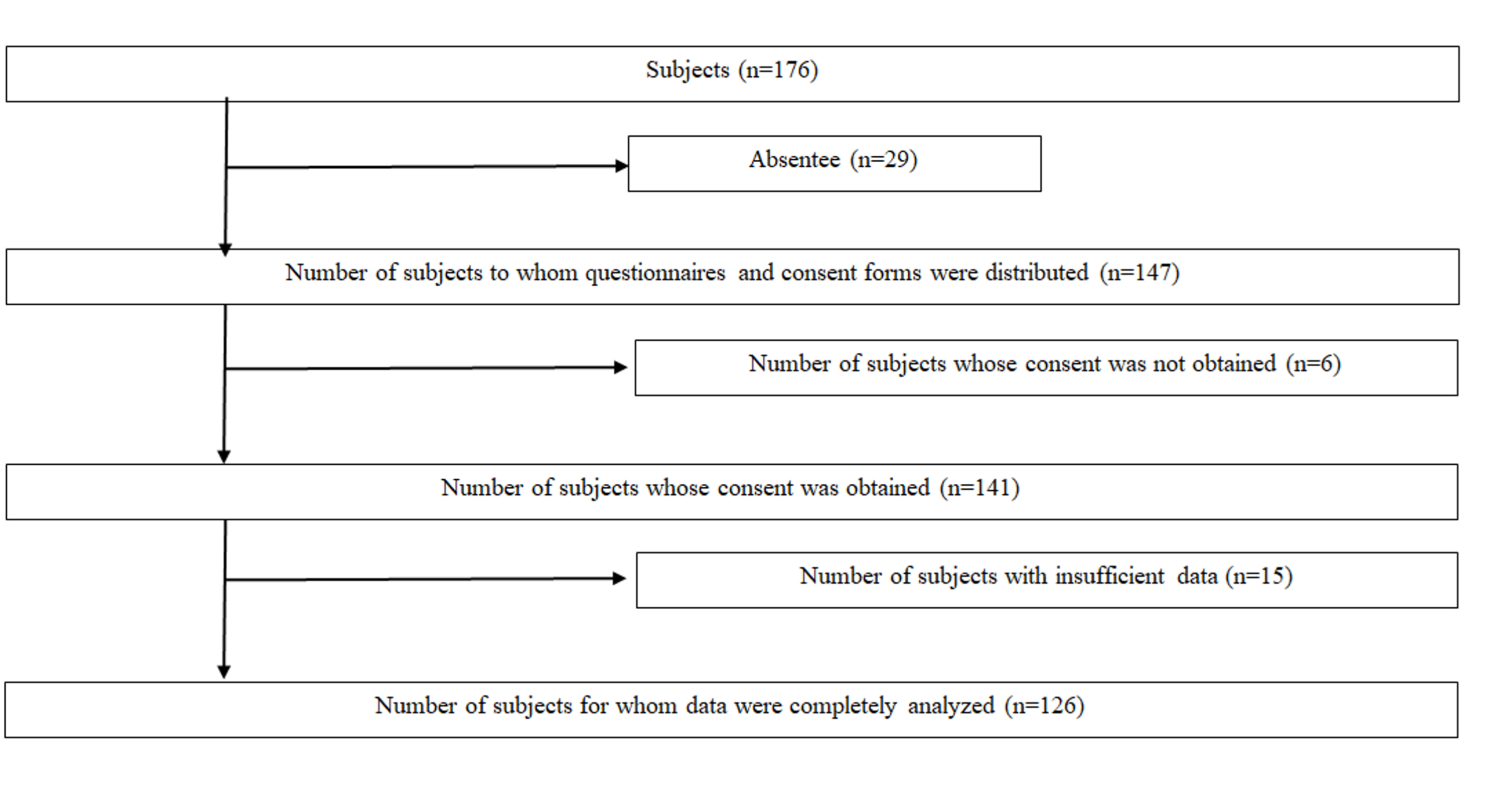
Flow chart for selecting subjects

A total of 126 (74 males and 52 females 19.1 ± 0.1 years old) of 176 first- and second-year educational faculty students at a university in Hyogo Prefecture, Japan, who met the following criteria were enrolled: (1) received and completed the questionnaire survey from September 22, 2025 to October 3, 2025, and (2) provided written informed consent (Figure 1). These grades were selected as they represent the early university years, a stage where experiences with sex education and personal sexual decision-making are still developing, making them an appropriate group for assessing basic knowledge about STIs. However, the study was conducted at one university. Therefore, finally, only 126 students were enrolled in this study.

Ethical approval was obtained from the Ethical Committee at the Research Ethics Review Committee, Faculty of Education, Kansai University of Welfare Studies (approval number: 7-0825-1, approval date: August 25, 2025).

Questionnaire

The questionnaire for this study was developed in Japanese based on prior research [13], approved by the affiliated institution, and administered using Google Forms®. The survey included open-ended questions such as “Please tell us what you know about AIDS. (Please answer in writing)” and “Please tell us what you know about syphilis. (Please answer in writing)”. We also investigated participants’ knowledge of the number of AIDS and syphilis patients in the past 10 years in Japan (increase/decrease), their sexual intercourse experience (yes/no), use of contraceptives for infection prevention (yes/no), and fears toward AIDS and syphilis (yes/no).

Statistical analysis

Data were expressed as the mean ± standard deviation and number of subjects (%). The chi-square test and Fisher’s exact test were used to examine differences in proportions between two groups, with p<0.05 being significant. These analyses were performed using JMP Student Edition 19 (SAS Institute Inc., Cary, NC).

Free-response data were analyzed using text mining software (KH coder®, KH Coder 3. Beta. 08e, Koichi Higuchi, Tokyo, Japan), as in previous studies [14,15], the reliability of which has been demonstrated [16]. We used KH coder® to create a list of frequently occurring words from the collected text and performed a co-occurrence network analysis [17] to reveal connections between words and word clusters. A co-occurrence network represents words frequently used together in analyzed text data using nodes (points or circles) and lines. The node size indicates the frequency of a word’s usage, while lines show words that are used together. By examining the words connected by these lines, we may obtain a more detailed understanding of the relationships between words and infer the general context of what is being discussed. Extracted words were translated into English using DeepL® translation [18].

## Results

The clinical characteristics of enrolled students are summarized in Table 1. The students recognized that AIDS and syphilis have both increased in the past decade, with no significant difference between sexes. However, the number of syphilis patients has increased while the number of AIDS patients has decreased during the same period, indicating a significant discrepancy between students’ perceptions and actual incidence trends (p <0.001).

**Table 1:** Clinical profiles of enrolled subjects Mean age (SD)*: 19.1 ± 0.1 *Standard deviation

		Number of subjects	%
Sex	Male	74	58.7
	Female	52	41.2
Age (years)	18	26	20.6
	19	62	49.2
	20	38	30.1

Fifty males (67.6%) and 28 females (53.9%) had sexual intercourse experience. Of these, 76% of males and 71.4% of females consistently used contraceptives. The percentage of males who consistently used contraceptives was higher than that of females. Furthermore, 64 males (86.5%) and 48 females (92.3%) expressed fears toward AIDS and/or syphilis (Table 2).

**Table 2:** Questionnaire responses by sex The chi-square test^a ^and Fisher's exact test^b^ were used to analyze data. AIDS, acquired immunodeficiency syndrome.

Question	Answer	Total	%	Male	%	Female	%	p^a^	p^b^
Which of the following applies to the number of AIDS patients in Japan in the last 10 years?	Increasing	101	80.1	58	78.3	43	82.7	0.55	
	Decreasing	25	19.8	16	21.6	9	17.3		
Which of the following applies to the number of syphilis cases in Japan in the last 10 years?	Increasing	109	86.5	66	89.2	43	82.7	0.29	
	Decreasing	17	13.5	8	10.8	9	17.3		
Have you ever had sexual intercourse before?	Yes	78	61.9	50	67.6	28	53.9	0.12	
	No	48	38.1	24	32.4	24	46.2		
If you answered "yes," do you use contraceptives during sexual intercourse to prevent infection?	Yes	58	74.4	38	76.0	20	71.4	0.65	
	No	20	25.6	12	24.0	8	28.6		
Have you ever been afraid of AIDS or syphilis?	Yes	112	88.9	64	86.5	48	92.3		0.39
	No	14	11.1	10	13.5	4	7.7		

We then extracted frequency words using a text mining analysis (Table 3). Text mining results on knowledge of AIDS and syphilis showed a total word count of 466 for AIDS and 325 for syphilis, indicating fewer total words for the latter (Table 4). The most frequent word was “infection,” followed by “sexually transmitted infection” for both diseases. The results of the free-response writing section showed that AIDS and syphilis were both frequently mentioned as individual words.

**Table 3:** Frequently used words for AIDS AIDS, acquired immunodeficiency syndrome.

Rank	Extracted word	Number of occurrences	%
1	Infection	38	8.2
2	Sexually transmitted disease	18	3.9
3	Illness	15	3.2
4	Sex	11	2.4
5	Person	8	1.7
6	Know	7	1.5
7	Immune	7	1.5
8	Heal	6	1.3
9	Blood	5	1.1
10	Sexual intercourse	4	0.9

**Table 4:** Frequently used words for syphilis

Rank	Extracted word	Number of occurrences	%
1	Infection	23	7.1
2	Sexually transmitted disease	21	6.5
3	Sex	14	4.3
4	Know	12	3.7
5	Illness	7	2.2
6	I understand	3	0.9
7	Sexual intercourse	3	0.9
8	Die	3	0.9
9	Negotiation	2	0.6
10	Person	2	0.6

**Figure 2:**
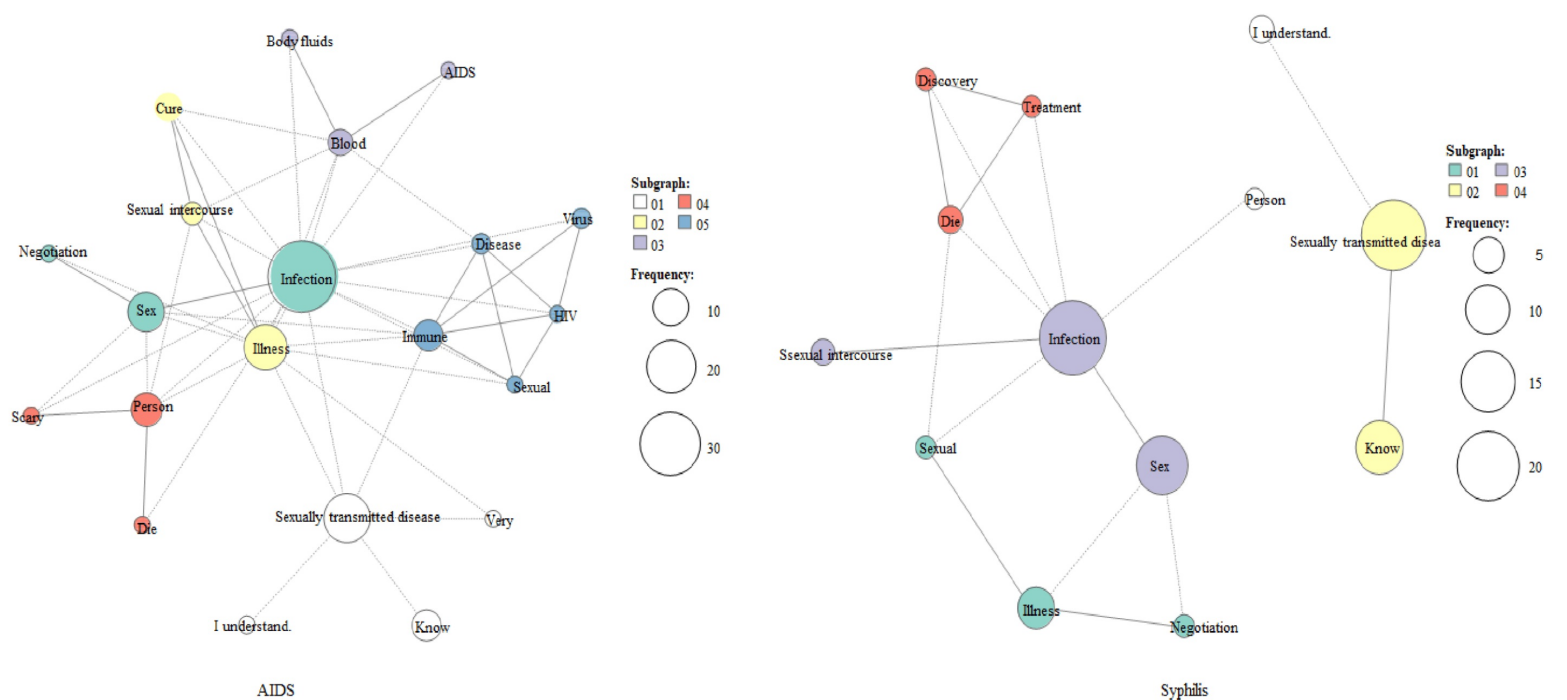
Co-occurrence network of AIDS and syphilis Node size indicates word frequency, and lines represent the relationship between co-occurring words. AIDS, acquired immunodeficiency syndrome.

An analysis using co-occurrence networks also revealed fewer word types and clusters for syphilis than for AIDS. Medical terms such as “immunity” and “HIV” were noted for AIDS, while medical terms related to syphilis were rarely observed (Figure 2).

## Discussion

The present study investigated knowledge of AIDS and syphilis in educational faculty students using a text mining analysis. The results obtained showed that knowledge of AIDS and syphilis, particularly syphilis, was poor among these students. Therefore, proper and basic STI education for these students is needed to prevent STI considering their future involvement in sex education. 

Kassie et al. reported that the probability of STI in Ethiopian university students was approximately threefold higher in students with less knowledge of STI than in those with proper knowledge [5]. Engaging in sexual intercourse without contraceptives and insufficient knowledge of STI were associated with infection [5]. Subbarao et al. investigated university students’ knowledge of STIs other than HIV, and showed that 90% of students had heard of STIs and 64% had heard of STIs other than HIV. Their findings also revealed that while 99% of students knew about HIV, less than 50% had knowledge of other STIs [6]. Tanimoto et al. showed that knowledge of HIV/AIDS among nursing students in Japan was high, and more than 50% had heard of chlamydia, herpes, and gonorrhea; however, limited knowledge of STIs other than HIV/AIDS was noted, with variations in knowledge depending on the disease [7].

In the present study, the text mining analysis showed that students had limited knowledge of AIDS and syphilis, particularly syphilis. Despite requesting written responses, most were single-word replies. Furthermore, many students were unaware of the number of AIDS patients in the past decade. Educational faculty students are expected to be involved in children’s sex education as future teachers. Therefore, their extremely low level of knowledge of AIDS and syphilis is a significant issue. It has become clear that the curriculum of the Faculty of Education must first provide basic knowledge of STIs, such as AIDS and syphilis. The present results also revealed that some students do not consistently use contraceptives (males: 24% and females: 28.6%), indicating a need to educate students on the proper use of contraceptives for themselves. Kubo et al. targeted undergraduate students in the Faculty of Education and conducted an intervention involving case-based discussions and personal life planning, such as STI, pregnancy, and infertility, building relationships with partners, and respecting diverse sexualities. The findings obtained showed an increase in correct response rates for knowledge of reproductive function, contraceptive methods, and STIs after the intervention [19]. However, in the study by Saito et al., while most students in the Faculty of Education considered group-based sex education to be necessary, they expressed anxiety and reluctance about actually teaching it themselves [4]. Kinkin et al. targeted undergraduate students in the Faculty of Education and revealed that the primary reasons for difficulties with sex education were “lack of specialized knowledge” and “uncertainty about teaching methods” [20]. Therefore, providing basic knowledge of STIs to first- and second-year educational faculty students may also help them to overcome their anxiety and hesitation when implementing sex education in the future.

There are a number of limitations that need to be addressed. The sample size was small, and the study involved a single university in Hyogo Prefecture, Japan. In addition, the students who were enrolled and responded were considered to be more health-conscious than the average student. Therefore, the results obtained herein may not apply to all educational students throughout Japan. Furthermore, since questions in the survey were about sex, they may not have been answered accurately. Loaiza-Guevara et al. point out that research related to STIs may be susceptible to the influence of social acceptance bias [21]. Nevertheless, the results obtained in the quantitative analysis using text mining provide valuable information on the knowledge of AIDS and syphilis among educational faculty students.

## Conclusions

We herein investigated knowledge of AIDS and syphilis among educational faculty students. The results obtained showed that knowledge of AIDS and syphilis was poor among these students and also that proper and basic knowledge of AIDS and syphilis, particularly syphilis, is urgently required among educational faculty students, considering their future involvement in sex education. 

## Appendices

Questionnaire Survey Content

Question 1 Please tell me your age. (Select)

 18 years old/19 years old/20 years old,

Question 2 Please indicate your gender. (Select)

 Male/Female

Question 3 Please tell us what you know about AIDS. (Please answer in writing)

Question 4 Please tell us what you know about syphilis. (Please answer in writing)

Question 5 Which of the following applies to the number of AIDS patients in Japan in the last 10 years? (Select)

increase/decrease

Question 6 Which of the following applies to the number of syphilis cases in Japan in the last 10 years? (Select)

 increase/decrease

Question 7 Have you ever had sexual intercourse before? (Select)

 Yes/No

Question 8 If you answered "yes", do you use contraceptives during sexual intercourse to prevent infection? (Select)

 Yes/No

Question 9 Have you ever been afraid of AIDS or syphilis? (Select)

 Yes/No
